# The Genetic and Hormonal Inducers of Continuous Flowering in Orchids: An Emerging View

**DOI:** 10.3390/cells11040657

**Published:** 2022-02-14

**Authors:** Sagheer Ahmad, Donghui Peng, Yuzhen Zhou, Kai Zhao

**Affiliations:** 1 Key Laboratory of National Forestry and Grassland Administration for Orchid Conservation and Utilization at College of Landscape Architecture, Fujian Agriculture and Forestry University, Fuzhou 350002, China; sagheerhortii@gmail.com (S.A.); zhouyuzhencn@163.com (Y.Z.); zhaokai@fjnu.edu.cn (K.Z.); 2 College of Life Sciences, Fujian Normal University, Fuzhou 350117, China

**Keywords:** continuous flowering, orchids, key regulators, hormones, miRNAs, transcription factors

## Abstract

Orchids are the flowers of magnetic beauty. Vivid and attractive flowers with magnificent shapes make them the king of the floriculture industry. However, the long-awaited flowering is a drawback to their market success, and therefore, flowering time regulation is the key to studies about orchid flower development. Although there are some rare orchids with a continuous flowering pattern, the molecular regulatory mechanisms are yet to be elucidated to find applicable solutions to other orchid species. Multiple regulatory pathways, such as photoperiod, vernalization, circadian clock, temperature and hormonal pathways are thought to signalize flower timing using a group of floral integrators. This mini review, thus, organizes the current knowledge of floral time regulators to suggest future perspectives on the continuous flowering mechanism that may help to plan functional studies to induce flowering revolution in precious orchid species.

## 1. Introduction

Flowering plants dominate the terrestrial landscape and, therefore, play a pivotal role in human life. Plant species adjust their flowering times using a fine combination of internal and external stimuli to adapt to the versatile environments [1]. Therefore, floral timing is one of the most important horticultural characteristics of floriculture crops, and a stable time of flowering is the main goal of breeding programs to induce horticultural novelty in commercial crops. Researchers have found a number of pathways that regulate floral timing and the signals generated by these pathways are integrated through floral integrators to synthesize a specific route of flowering initiation [2]. Five pathways have been recognized in Arabidopsis to control flowering, including photoperiod, vernalization, autonomous, aging and gibberellin pathways [3]. The key integrators perceiving these pathways include *FT* (*FLOWERING LOCUS T*), *FD* (*FLOWERING LOCUS D*) and *SOC1* (*SUPPRESSOR OF OVEREXPRESSION OF CONSTANS*) [3,4,5]. Flowering signals are transmitted by these integrators to the floral meristem identity genes, such as *LFY* (*LEAFY*) and *AP1* (*APETALA1*). The activation of meristem identity genes is followed by floral development in association with MADS-box genes and co-regulators [1,5].

Orchidaceae contains the most important ornamental flowers [6,7], and that is why more than 0.1 million orchid species are cultivated throughout the world [1]. Such a beauty waits a long time to flower. *Phalaenopsis*, *Cymbidium* and *Dendrobium* are among the most attractive flowers, which bloom in specific seasons [8]. However, there is an orchid species, *Arundina graminifolia*, which gives continuous flowering throughout the year unlike other orchids [1]. Finding the key regulators of this continuous flowering pattern can provide a valuable source to find applicable solutions to seasonal flowering orchids. Therefore, this review focuses on the currently identified molecular regulators of the continuous flowering pattern in orchids and other species and the future perspective of this knowledge to modify seasonal flowering orchid species to boost short vegetative phases and recurrent flowering. 

## 2. Genetic Regulators of Flowering Time

Several MADS-box genes have been identified in different orchid species owing to their putative roles in flower development [9,10]. *Doritaenopsis* hybrid photoperiod regulated *EFL2* (*EARLY FLOWERING*-like), *DhEFL3* and *DhEFL4* delay flowering when overexpressed in Arabidopsis [11]. *Doritaenopsis* ortholog of Arabidopsis *FVE* (*DhFVE*) regulates flowering in the autonomous pathway [12]. *A CONSTANS*-like gene from *Phalaenopsis* (*PhalCOL*) induced early flowering when expressed in tobacco [13]. *GIGANTEA (GI)* is an upstream activator of *CO* [14]. In *Doritaenopsis* hybrid, *DhGI1* has been identified as having an important role in flowering initiation [15]. Moreover, CDF (CYCLING DOF FACTOR) and FKF1 (GI-FLAVIN-BINDING, KELCH REPEAT, F BOX 1) also regulate the activity of *CO* in *Phalaenopsis* [16]. The ectopic expression of *P. aphrodite FT1* (*PaFT1*) showed precocious flowering in rice and Arabidopsis [17]. Moreover, expression of *PaFT1* in Arabidopsis phloem suppressed the late flowering caused by *FRIGIDA* (*FRI*) allele and overexpressed *SHORT VEGETATIVE PHASE* (*SVP*) [18]. *PaFD* is a bZIP domain-containing transcription factor that is considered to be a PaFT1-interacting protein. It can partially complement the late flowering phenotype of Arabidopsis fd-3 [19]. The *Oncidium* Gower Ramsey *TERMINAL FLOWER 1* (*OnTFL1*) and *FT* (*OnFT*) play antagonistic roles to regulate flowering in Arabidopsis [20]. *P. aphrodite LEAFY* (*PhapLFY*) accumulates in the primordia of floral meristem to induce flowering initiation [21]. *DOH1* (*DENDROBIUM ORCHID HOMEOBOX1*) is downregulated during floral transition in the shoot apex as an upstream regulator of *DOMADS1* expressed during the transition of shoot apical meristem, thereby advancing the flower transition and development [22]. No *FLC* homologs have been documented in sequenced orchid species [23], but homologs of Arabidopsis *AGL19* are identified in *D. nobile*. *FLC* is an important vernalization pathway gene for flowering regulation. The absence of *FLC* homologs in the orchids, such as *C. sinense*, *P. aphrodite* and *D. catenatum*, can be compensated by the presence of *VRN*-driven vernalization responses independent of *FLC* [23]. Moreover, the expression of *OMADS1* (*O. MADS1*) of *AP1*/*AGL9* of MADS-box genes has been detected in the apical meristem and reproductive parts of flowers. *OMADS1* also regulates the floral initiation of *O.* Gower Ramsey in association with *OMADS3* [24]. The ectopic expression of *OMADS1* homolog *AGL6* caused early flowering in Arabidopsis [24]. *P. equestris DEF*-like genes, *PeMADS2*, *PeMAD3*, *PeMADS4* and *PeMADS4*, have been identified in floral organs, suggesting a role in flowering control [25].

## 3. Important Transcription Factors

**Figure 1:**
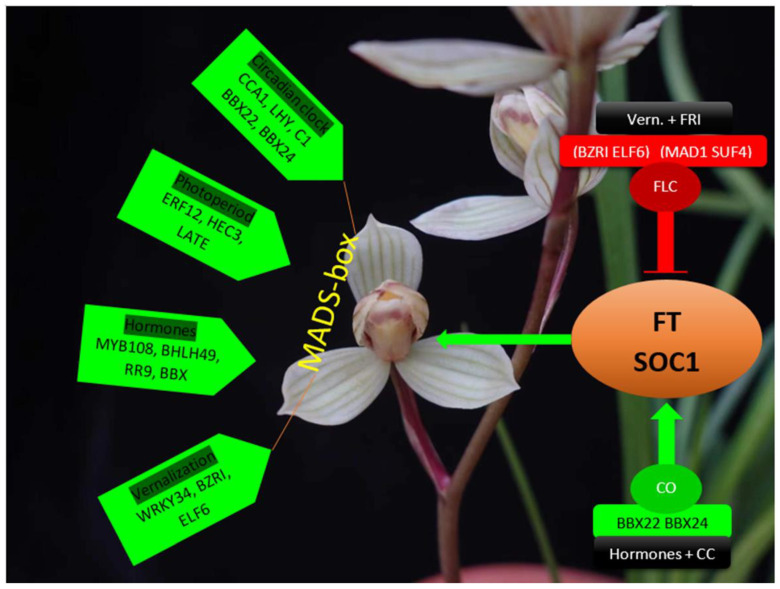
The summary of important transcription factors in the regulation of flowering in orchids. Most of these transcription factors have been identified in continuous flowering orchid *A. graminifolia* [1,73].

More than 35 transcription factor families have been identified in *A. graminifolia* that may play roles in controlling continuous flowering [26]. In the photoperiodic pathway, HECATE3 (HEC3) regulates phytochrome signaling and together with LHY, CCA1 and CO-like transcription factors it controls flower development (Figure 1) [26]. A number of TFs interact with gibberellic acid (GA), and bHLH and ERF intervene in flowering through auxin and ethylene signaling pathways, respectively [27]. Auxin and ethylene may act synergistically or antagonistically with GA to control flowering [28,29,30,31]. VP1 (Viviparous-1) acts in the abscisic acid (ABA) pathway by encoding a B3 type transcription factor [32]. 

Four important transcription factor families, including MYB, ZFP, bHLH and WRKY, are known for flowering regulation in many plant species [27]. In Arabidopsis, some WRKY TFs, such as WRKY12, 13 and 17, MYB TF EFM (EARLY FLOWERING MYB PROTEIN), and bHLH members, such as bHLH48 and bHLH60, involve flowering regulation through FT transcription [33,34,35,36]. In addition, MADS-box transcription factors, such as *AGAMOUS-like 5* (*AGL5*), *AGL6*, *MADS14*, *MADS16*, *APETALA3* (*AP3*) and *SEPALATA* (*SEP*) are thought to regulate continuous flowering in *A. graminifolia* [26,37,38]. The *AGL6* knockdown by artificial miRNA caused late flowering, where its activation by *35S*enhancer stimulated early flowering [39]. In *O.* Gower Ramsey orchids, two *AGL6-like* genes, *OMADS1* and *OMADS7* have been identified with their overexpression leading to early flowering in Arabidopsis [40,41], suggesting the conserved role of *AGL6-like* genes in flowering time regulation. AP1 is a MADS-box protein controlling floral meristem identity, and the overexpression of *AP1* causes early flowering [39]. *AP1* orthologs have been identified in orchids, such as *Cymbidium*, *Oncidium* and *Dendrobium* [39]. *DOMADS2,* an *AP1-*like gene in *Dendrobium* Madame Thong-In, expresses during floral transition [42]. Similarly, *Erycina pusilla AP1-*like gene, *EpMADS12,* involves floral organ development [43]. Overexpression of *AP1* orthologs, *OMADS10* and *DOAP1*, caused early flowering in Arabidopsis [41,42]. Moreover, the overexpression of *DOAP1* in *Dendrobium* accelerated flowering as compared to wild types [42].

MADS14 is an AP1/FRUITFUL (FUL)-like MADS-box TF that involves meristem identity [44,45,46]. *MADS14* and *MADS16*, an *AP3*-*PI* subfamily genes, and *SEP* were highly expressed in the early stages of flower development of *A. graminifolia* [1,26]. In *Phalaenopsis* hybrid Athens, *SEP*-like genes (*PhaMADS4*, *PhaMADS5* and *PhaMADS7*) express in floral organs [47]. Four *SEP*-like genes (*PeSEP*1–4) have been identified in *P. aphrodite* [48]. TCP3, a transcriptional activator of CO [49], also showed high expression in the early stages of orchid flower development. CIRCADIAN CLOCK ASSOCIATED1 (CCA1) represses *GI* and *SOC1* and regulates flower initiation and development of orchids [50]. 

MYB108 acts in the jasmonate-mediated pathway for stamen maturation and plays a pivotal role in correctly deciphering the timing of anther dehiscence. It regulates pollen viability in association with MYB24, and its expression is also controlled by upstream MYB21 [51]. EAT1 (ETERNAL TAPETUM1) is a bHLH TF that regulates tapetal cell-fate decision [52]. It is specifically expressed to stimulate floral initiation in *A. graminifolia*. BHLH49 controls auxin regulation for embryonic identity [53], while RR9, a type-B response regulator, involves cytokinin signaling. WRKY34 regulates vernalization-mediated flowering through proteolysis of FRI [54]. ERF12 (ETHYLENE RESPONSE FACTOR 12) integrates AP2 to control meristem identity for flower initiation and floral timing [55,56].

Flower development requires complex transcriptional regulation using the associated roles of zinc finger transcription factors (ZFPs), MYBs, bHLHs, MADS-box and the DNA-binding domains [57,58,59,60]. Zinc finger is a pivotal domain among the transcription factors [61]. C2H2 ZFPs are known for their important roles in the floral induction, hormonal regulation and cell division and proliferation [62]. They are thought to transcriptionally control flowering through the chromatin modification of FLC, wherein C2H2-ZFs cause the histone modification of FLC to induce flowering. They also act downstream of AP1 and interact with miR164 to regulate flowering [1]. C2H2-ZFPs participate in the photoperiodic pathway of FT, and also involve the histone modification of FT locus [62]. CO, the core integrator of the photoperiodic regulation of its downstream genes *FT* and *SOC1*, is a B-box zinger binding domain containing protein (Figure 1) [63,64]. Three *CO*-like genes have been shown to regulate flowering time in *C. ensifolium* [65]. Circadian clock coordinates some B-box proteins in the photoperiodic regulation of flowering [66], while it also controls CO expression [67,68]. HUA1, a CCCH-type ZFP, in association with AGAMOUS (AG) regulates flowering through its downstream genes [69]. 

ELF6 activates FLC through H3K27 demethylation [70] and by interaction with BZR1 (BRASSINOZOLE-RESISTANT 1) [62]. SUF4 is another zinc finger protein that can positively regulate FLC by FRI [71,72]. Mitotic arrest deficient 1 (MAD1) is a mitotic spindle checkpoint zinc finger protein that positively regulates FLC [73]. The MAD1 and SUF4 interaction can regulate floral timing [62]. RBE, encoding a C2H2 ZFP, acts downstream of AP1 to regulate flowering [74]. LATE controls the expression of FT in the photoperiodic pathway [73]. Its expression in leaf vascular tissues inhibits the FT response in the long days [75]. High LATE expression was observed in the early stages of flower development in *A. graminifolia* [73]. Knuckles (KNU), encoding a SUPERMAN-like protein, involves floral regulation in association with AG [76]. HUA1 is very important CCCH-type ZFP in the meristem determinacy [69,77]. 

CO is the core photoperiodic regulator, upregulating its downstream genes FT and SOC1 [63]. It is a B-box (BBX) binding domain containing zinc finger protein [64]. All the BBX proteins need CO to regulate flowering (Figure 1). BBX24 is an important regulator of flowering [78,79,80,81]. It regulates GA biosynthesis and photoperiodic pathway genes to control flowering time in chrysanthemum [82]. Another B-box ZFP BBX22 acts in the photomorphogenesis [66,83,84,85,86]. BBX22 also regulates ARR10 to mediate cytokinin responses [87]. Both BBX22 and BBX24 show distinct responses in the circadian rhythm pathway [66] and ABA application [88], while BBX24 can also regulate flowering time independent of CO [79,80] by repressing FLC and activating FT and SOC1 in independent events. BBX24 may also instigate flowering time through brassinosteroid and ethylene [79], and it has a well-elucidated role in auxin, GA and ABA signaling [89,90,91,92,93,94]. 

## 4. The miRNAs Controlling Flowering Time

**Figure 2:**
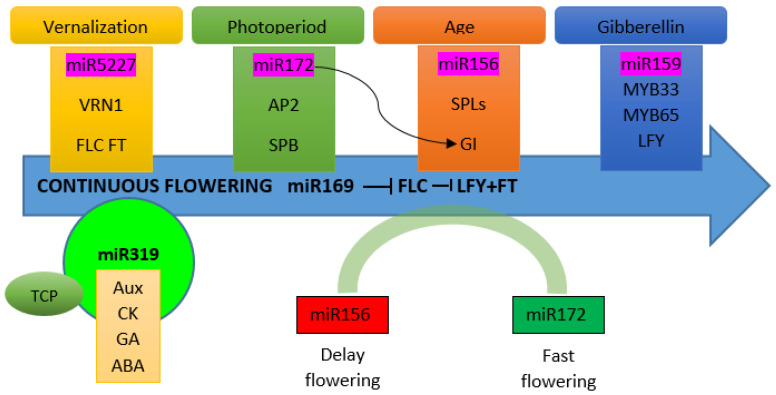
Proposed key miRNAs that may play a role in continuous flowering regulation in orchids.

A number of studies suggest the role of important miRNAs playing roles in flowering regulation [95,96]. In short days, miR159 regulates flowering [97,98,99]. Squamosa promoter binding (SPB) genes involve circadian clock regulation of vegetative to reproductive phase transition [100,101,102]. Some SPB genes are regulated by miR156 and miR172 in the regulation of flowering [103,104]. Overexpression of miR156 suppresses SPB, thereby delaying flowering [105], while miR172 overexpression accelerates flowering [106,107,108]. Thus, the role of miR156 and miR172 is antagonistic in floral regulation (Figure 2) [109,110]. While switching from the vegetative stage to reproductive stage, the expression of miR156 decreases, while that of miR172 increases [102,106,107,111]. This antagonistic role of miR156 and miR172 has been observed in the orchid *P. aphrodite* [112]. Moreover, miR172 targets AP2 to regulate floral organ identity [106,108]. The *C. ensifolium CeAP2-*like gene is a potential target of miR172 [113]. The miR172 also interacts with GIGANTEA (GI), which is a flowering regulator in the circadian clock pathway [114]. In the GI mutant, miR172 expression was significantly decreased, while it remained unaffected in CO mutant [107], suggesting that GI-mediated floral regulation by miR72 is independent of CO. The module of miR-156 and miR172 is conserved for its role during the phase transition from the vegetative to reproductive phase in orchid species, such as *P. aphrodite* [115], *Orchis italica* [116] and *E. pusilla* [117].

Overexpression of miR169 suppressed the expression of FLC, which is a key repressor of flowering, allowing the increase in the expression of FLC targets, *LFY* and *FT* [118]. Thus, the regulatory pathway of miR169 is different from the miR156-miR172 module [119]. In Arabidopsis, miR319 targets TCP transcription factors (Figure 2) [120] and the overexpression of miR319 suppresses the expression of TCP genes, suggesting a role in floral timing [118]. The miR319 regulates flower development of the orchid *O. italica* by targeting TCP proteins [121]. 

MYB genes regulating flower development are targeted by miR159 (Figure 2) [122,123]. The miR159 causes the downregulation of *LFY* through degradation of MYB33 in GA-induced pathway of flowering regulation in Arabidopsis, causing the delay of flowering in short days [97,98,99]. The miRNA studies in *P. aphrodite* show that mirRNA159 targets the MYB family and the miRNA319 targets the TCP family [112]. 

The miR319 and miR396 possess a wide range of interactions with phytohormones [124]. Auxin signaling is positively regulated by miR319 [125,126], while cytokinin is negatively regulated, suggesting the important role of this miRNA in the antagonistic auxin-cytokinin module [127,128]. The miR319 also inhibits GA biosynthesis, thereby affecting cell differentiation in Arabidopsis [129,130]. Similarly, it may also play role in the antagonistic ABA-GA pathway, as miR319 expression was suppressed by ABA treatment in rice [127]. However, ABA supports the biosynthesis of miR159 and miR393 [124,131]. Therefore, miR159 is a valuable link between GA, ABA and ethylene. Moreover, in Arabidopsis, GA promotes flowering through miR156 [104]. 

Floral time control has been shown by miR159, targeting MYB TFs in the GA pathway [132]. Application of GA degrades the DELLA protein, causing the increase in miR159 level [133,134,135]. The role of flowering time manipulation by the miR159-MYB module has been documented in *A. thaliana* [136] and radish [132]. In radish, miR159 targets two MYB genes, MYB65 and MYB101. Moreover, two radish miRNAs, miR824 and miR5227, target two flowering-related MADS-box TFs, AGL16 and VRN1, respectively [132]. In Arabidopsis, AGL16 modulates flowering time through its interaction with FLC, SVP and FT [137], while VRN1 represses the floral inhibitor FLC [138] and facilitates the rhythmic modulation of FT [139]. 

The miRNA studies in *P. aphrodite* suggested that miRNA156 targets SPL genes, miR159 targets MYB genes, miRNA167 targets auxin response factors, while the miRNA172 targets AP2 genes [112]. In *D. catenatum*, the miR156 also targets SPL genes [140].

## 5. Hormonal Regulators of Flowering Time

Plant hormones are always important for the flowering regulation of orchids [141]. Auxin is a morphogen [142,143,144,145,146] and signalizes tissue specification based on its concentration gradient [147]. The application of 6-benzylaminopurine, a synthetic cytokinin, promotes flowering in *Dendrobium* and *Phalaenopsis* orchids, but auxin antagonizes this effect. In *Doritaenopsis* and *Phalaenopsis,* exogenous BA application promotes early flowering [148]. Interestingly, although GAs do not induce flowering, optimum accumulation of GAs is required inside the shoot tips to support flower development in *Phalaenopsis* [149]. Injecting GAs can restore the blockage of flower development of *P. hybrida* due to high temperatures [150]. BA applied in combination with gibberellin (GA3) makes a pronounced effect on flowering [151]. GA controls important processes, such as stem elongation and flowering time [152,153,154,155,156], whereas ABA is a regulator of flowering time and bud break [157,158]. Strigolactone are thought to play roles in flowering regulation through their cross-talks with GA, ethylene, auxin, and cytokinin [159,160,161]. 

**Figure 3:**
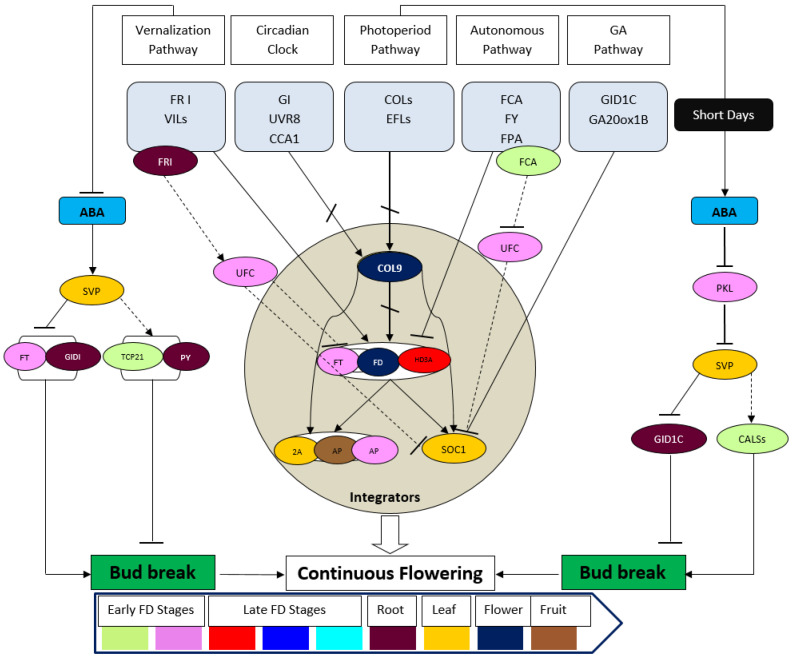
Hypotheses on multiple pathway regulation of continuous flowering in orchids, specifically the *A. graminifolia*, independent of TFL1. It may serve as a basis to study the phenomenon of continuous flowering in seasonal orchids. The color of circles shows the observed expression of different pathway genes in *A. graminifolia* [1].

ABA involves bud dormancy control in the photoperiodic pathway [162,163,164]. ABA upregulates CALLOSE SYNTHASE 1 (CALS1) (Figure 3) and represses glucanases, causing the blockage of intercellular conduit (plasmodesmata) using dormancy sphincters (callosic plugs) that hinder growth promoting signals to promote dormancy [163]. A number of CALS homologs were observed in the transcriptome of *A. graminifolia*. CALS1 was highly expressed in early stage of floral bud outgrowth. Moreover, ABA-responsive ABFs showed high expression in early bud stages, suggesting temporal control of bud development mediated by ABA [1]. The concentration of ABA has been examined in different tissues of *Phalaenopsis*, and high amounts of free ABA were found in dormant axillary buds [165]. Moreover, the exogenous ABA application to *Phalaenopsis* stem inhibits the floral spike formation, suggesting the inhibitory role of ABA in orchid floral transition [149]. However, detailed functional studies will be needed to fully understand the role of ABA in flowering control for orchids. 

## 6. Continuous vs. Seasonal Flowering

Continuous flowering (CF) is the most economically important horticultural trait for orchids, although little progress is made to understand and apply this phenomenon. It not only affects flowering habit, but also causes important development changes, such as short juvenile phases and rapid flowering after germination from seed [166,167]. In roses, CF is regulated by TERMINAL FLOWER 1 (TFL1) gene family [168]. TFL1 controls the inflorescence identity of shoot apical meristem in a number of crops [169,170,171,172,173], such as pea [174,175], and *A. thaliana* [176,177]. TFL1 also affects the length of vegetative phase in Arabidopsis [174,176].

Other than non-model species [178,179,180], the*TFL1*-like genes have been studied in the Orchidaceae, revealing their functional diversity in different orchid species [181]. The *Cattleya trianae CatrTFL1* shows broad expression patterns in various tissues, and the *Gomphichis scaposa GoscTFL1* shows expression in floral buds and SAM [182]. *D. catenatum DcHd3b* highly expresses in the seedlings during juvenile growth [140]. 

In *Dendrobium* Orchid, *TFL1* ortholog (*DOTFL1*) regulates floral transition and its ectopic expression rescues early flowering in Arabidopsis, while its overexpression delays flowering [181]. Therefore, *DOTFL1* is essential to regulate flowering time and flower development in *Dendrobium* orchids [181]. *OnTFL1*, a *TFL1* ortholog in *O.* Gower Ramsey orchid, suppresses floral transition [20]. Interestingly, *TFL1* genes were not observed in the transcriptome of *A. graminifolia*, suggesting that the routes of continuous flowering regulation are not facing checks like in other orchids by *TFL1*. *TFL1* also interacts with FD, thereby negatively regulating the *LFY* and *AP1*. This interaction maintains a negative feedback loop between the floral meristem identity genes *LFY* and *AP1* and the inflorescence regulator *TFL1* during the vegetative-to-reproductive phase transition [183]. In *D. nobile*, orthologs of *FT* and *MOTHER OF FT AND TFL1* (*MFT*) have been identified to play role in flowering control [184].

FT/TFL1 gene family makes a pivotal regulatory network of flowering regulation. Changes in critical sites of amino acids lead distinct variations in the protein functions, although TFL1 and FT show high amino acid identity (~60%) [185]. FT promotes flowering, its transcripts move from leaf to shoot apical meristem (SAM) through phloem while physically interacting with FD protein [44,46,185]. Homologous genes of FT have been identified in several orchid species, including *Dendrobium* [184], *C. faberi* [186], *Oncidium* [20], *C. goeringii* [187,188], *Phalaenopsis* [17,189] and *C. sinense* [23]. Moreover two FT-like genes have been cloned from *A. graminifolia* [1] and one in *P. aphrodite* [17,189] with predicted role in flowering regulation. In the orchids, such as *Cymbidium*, *Dendrobium* and *Oncidium*, the *FT* expression was mainly found in leaf and axillary buds and it was influenced by daylength in *Cymbidium* and *Oncidium* [20,109]. Moreover, the ectopic expression of orthologs of *FT*, such as *DnFT*, *CeFT*, *CsFT*, *OnFT*, *CgFT*, *DOFT*, and *PaFT* resulted in precocious flowering in transgenic plants [21,39,184,187,188,190,191]. Interestingly, in *Dendrobium* orchids, the downregulation of *DOFT* delays flowering, whereas its overexpression accelerates flowering [39,191]. Other than orchids, FT genes have been identified in a number of species [192,193,194,195,196,197,198,199,200,201]. 

The mechanism of continuous flowering is tightly linked to dormancy and bud release. Theories suggest that increase in the expression of FT/FD and GA biosynthesis genes induce bud release [202,203,204]. However, ABA antagonize this effect either by regulating GA levels through inhibition of SVP during short days, or downregulating FT at low temperature (Figure 3) [1]. 

*SVP* acts as a regulator of flowering time and positively regulates TCP18, which mediates bud break depending on temperature [205]. SVP and TCP18 make a transcriptional module sensitive to temperature to control bud break. *C. goeringii* SVP2 makes a loop with *CgSOC1* and *CgAP1*, forming the basis of MADS-box TF function (64), and AP1 serves as a hub between SVP and SOC1 to form flower induction pathway and interacts with floral organ identity proteins [37]. Overexpression of *Dendrobium* orchid *SOC1*, *DOSOC1*, caused early flowering both in *Dendrobium* and Arabidopsis [152]. SVP also regulates flowering by interacting with FLOWERING LOCUS M (FLM) and FLC in the temperature and photoperiod pathways [206,207,208]. The *Cymbidium* orchids have been reported to contain *SVP* orthologs, whereas no *FLC* homologs have been documented. The *SVP* expression is greatly affected by cold treatment in *C. goeringii* [8]. Moreover, SVP also targets ABA and GA pathway genes to regulate bud break [209]. However, it was almost undetectable during the flower development of *A. graminifolia*, although it was detected during early stages of flower development of *C. goeringii* [26]. 

Unlike seasonal flowering orchids, such as *C. sinense* and *P. aphrodite*, the *A. graminifolia* flowers throughout the year. Recent studies have identified a number of key floral regulators, such as *FT*, *SOC1*, *ELF*, *COL5*, *COL9*, key hormonal regulators of ABA and GA, and the autonomous pathway regulators (*FY*, *FCA* and *FPA*) (Figure 3). These, along with circadian clock agents (*GI* and *CCA1*), can make multiple regulatory conduits that may drive continuous flowering in *A. graminifolia*. ABA may use alternative pathways to regulate bud break through SVP in the vernalization and photoperiodic pathways (Figure 3). However, these assumptions are yet to be verified through a series of experiments to apply the knowledge to other seasonal orchid species. 

## 7. Conclusions

Flowering regulation is an intricate process involving multiple pathways regulated by intrinsic and extrinsic stimuli. Long juvenile phases of orchids are a challenge for the researchers to achieve continuous flowering that can not only accelerate the market success of orchid flowers but also bring an ornamental revolution. Although *A. graminifolia* is a rare orchid with a continuous flowering characteristic, the basic knowledge of key genetic regulators of this species can set a direction to genetically modify the precious orchid species to achieve flowering throughout the year. Therefore, this review shows that genetic integrators, MADS-box genes, miRNAs and transcription factors are required to perform through multiple pathways to regulate continuous flowering in orchids. The model of floral regulators in *A. graminifolia* (Figure 3) can be adopted as a basis to plan and direct future research for seasonal orchid species.

## Data Availability

This article does not generate any supplementary data.
